# Not your grandparents' cardiac care: The future of geriatric cardiology

**DOI:** 10.1002/clc.23270

**Published:** 2019-10-03

**Authors:** Nanette K. Wenger, Karen P. Alexander

**Affiliations:** ^1^ Cardiology Emory University School of Medicine Atlanta Georgia; ^2^ Cardiology Duke University Durham North Carolina

1

During the last 50 years, a tremendous evolution of medical, scientific, and technologic advances has coincided with the successful aging and expansion of the older US population. As a result, the current cardiovascular care of older adults differs vastly from the care of similarly aged patients in prior eras. Our grandparents would scarcely recognize the systems of care and treatment options available today. The use of the computer, internet, and smart phones has skyrocketed in parallel among today's older adults.^1^ Currently, one in four older adults are smartphone users, but the next wave of older adults will likely all own smartphones. Age‐adjusted heart disease deaths in the United States have declined between 1950 and 2010 with the population living longer and dying less often of acute cardiovascular events.^2^ These co‐occurring phenomena have brought about dramatic changes in how long we live, how well we live in later years, and even how we die.

For frame of reference on how far we have come, we reviewed the agenda and discussions from the 18th Bethesda Conference: Cardiovascular Disease in the Elderly held in 1987.^3^ Thirty years ago, the “elderly” referred to those aged ≥65 years—the Medicare age group. The physician's obligation was to provide the best possible care to the elderly patient—based on scientific knowledge, personal skills, and humanistic and reasonably efficient principles. There were ethical and moral concerns associated with the application of new diagnostic and therapeutic technology, and talk about possible government actions to control health care costs for an aging population. The approach to care was disease‐based, not patient‐centered. Although physiologic age was clearly emphasized, there was little knowledge about aspects of cardiovascular care over age 75. At that time, surgical valve replacement was the only option for severe calcific aortic stenosis, heart failure had a dismal prognosis, and the only device implanted was a pacemaker. The guiding principles for care of older adults were quality, access, and cost‐effectiveness.

In the contemporary era, an older adult is ≥75 years, and ≥ 65 is more late‐middle age. Many other constructs are similar. Although underrepresentation in research studies has been a concern,^4^ clinical trials are now conducted exclusively in older patients.^5^ Pragmatic methods are helping to align research with practice so that older adults are more easily included.^6^ Age‐friendly health systems are aligning care with what matters most to older adults as a top priority.^7^ The possibilities for improving healthy life years for older adults with evidence‐based high value cardiovascular care have never been more plentiful. The societal expectation guiding care for older adults includes new approaches with a focus on functional assessment, shared decision‐making, patient preferences, de‐prescribing and awareness of geriatric syndromes [Correction added on 8 February 2020, after first online publication: The preceding sentence has been updated. The sentence now includes "a focus functional assessment."].

So today, and in this issue of the Journal, our emphasis is returning the patient, rather than the disease, to center stage. Although the Clinical Practice Guidelines have provided a valuable resource for clinicians over the decades, the tailoring of Guideline recommendations to the preferences of the individual is a central theme. The development and honing of communication skills is an integral component of lifelong teaching and learning both in the clinical arena and in academia. Therefore, it is with pleasure and gratitude that we acknowledge the mentoring of our fellow‐in‐training and/or early career faculty manuscript authors by their senior faculty colleagues. We submit this collection of articles with a sense of reflection on how far we have come and with excitement on where we will go in the future.

## CONFLICT OF INTEREST

The authors declare no potential conflict of interest.

## References

[1] Pew Research Center Internet and Technology [Last accessed on August 28, 2019]. https://www.pewinternet.org/2017/05/17/technology-use-among-seniors/

[2] Mortality Trends in the United States 1900 – 2015. https://www.cdc.gov/nchs/data-visualization/mortality-trends/index.html

[3] Wenger NK , Marcus FI , O'Rourke RA . Cardiovascular disease in the elderly. J Am Coll Cardiol. 1987;10:80A‐87A. 10.1016/s0735-1097(87)80456-4 3598020

[4] Rich MW , Chyun D , Skolnick AH , et al. Knowledge gaps in older adult populations: a scientific statement from the American Heart Association, American College of Cardiology, and American Geriatric Society: executive summary. J Am Geriat Soc. 2016;64(11):2185‐2192. 27673575 10.1111/jgs.14576

[5] McNeil JJ , Nelson MR , Woods RL , et al. Effect of aspirin on all‐cause mortality in the healthy elderly. N Engl J Med. 2018;379:1519‐1528. 30221595 10.1056/NEJMoa1803955 PMC6433466

[6] Ford I , Norrie J . Pragmatic trials. N Engl J Med. 2016;375:454‐463. 27518663 10.1056/NEJMra1510059

[7] Age Friendly Health Systems. https://www.johnahartford.org/grants-strategy/current-strategies/age-friendly/

[8] Wenger NK. Do we need an Apgar score for older adults? *Circulation*. 2019; 140: 973‐975. [Correction added on 8 February 2020, after first online publication: Citation 8 added]. 10.1161/CIRCULATIONAHA.119.039670 31525104

